# Whole-slide image analysis identifies a high content of Hodgkin Reed-Sternberg cells and a low content of T lymphocytes in tumor microenvironment as predictors of adverse outcome in patients with classic Hodgkin lymphoma treated with ABVD

**DOI:** 10.3389/fonc.2022.1000762

**Published:** 2022-10-20

**Authors:** Antonio Santisteban-Espejo, Irene Bernal-Florindo, Jose Perez-Requena, Lidia Atienza-Cuevas, Nieves Maira-Gonzalez, Marcial Garcia-Rojo

**Affiliations:** ^1^ Department of Pathology, Puerta del Mar University Hospital, Cadiz, Spain; ^2^ Institute of Research and Innovation in Biomedical Sciences of the Province of Cadiz (INiBICA), Cadiz, Spain; ^3^ Department of Medicine, Faculty of Medicine, University of Cadiz, Cadiz, Spain; ^4^ Department of Pathology, Jerez de la Frontera University Hospital, Cadiz, Spain; ^5^ Department of Pathology, Puerto Real University Hospital, Cadiz, Spain

**Keywords:** whole-slide imaging, classic Hodgkin lymphoma, digital image analysis, tumor microenvironment, Hodgkin Reed-Sternberg

## Abstract

Classic Hodgkin lymphoma (cHL) constitutes the most frequent lymphoma in young adults. Its histopathology is unique as a scattered tumor population, termed Hodgkin Reed-Sternberg (HRS) cells is diluted in a prominent tumor microenvironment (TME) composed of T lymphocytes, B lymphocytes, macrophages, neutrophils, eosinophils and histiocytes. Traditionally, the identification of prognostic biomarkers in the cHL TME has required visual inspection and manual counting by pathologists. The advent of whole-slide imaging (WSI) and digital image analysis methods could significantly contribute to improve this essential objective in cHL research, as a 10-20% of patients are still refractory or relapsed after conventional chemotherapy. In this work, we have digitized a total of 255 diagnostic cHL slides and quantified the proportion of HRS cells (CD30), B cells (CD20) and T cells (CD3) by digital image analysis. Data obtained where then correlated with the overall survival (OS) and progression free survival (PFS) of cHL patients. Quantification of HRS cells, B cells and T cells reflects the biological heterogeneity of the different cHL histological subtypes analyzed. A percentage of 2.00% of HRS cells statistically significantly discriminated between patients achieving a complete metabolic response (CMR) and refractory or relapsed (R/R) patients both for the OS (*P*=0.001) and PFS (*P*=0.005). Furthermore, patients with a percentage of T cells below the 26.70% in the TME showed a statistically significantly shorter OS (*P*=0.019) and PFS (*P=0.041*) in comparison with patients above this threshold. A subgroup of patients with a low content of T cells and high content of HRS cells exhibited a special aggressive clinical course. Currently, there is the need to implement quantitative and easy scalable methods to enhance clinical translation, as the cHL TME plays a central role in the clinical course of the disease. The results of this study could contribute to the identification of prognostic biomarkers specifically looking at the cHL TME and their inclusion in future clinical trials.

## Introduction

Classic Hodgkin lymphoma (cHL) constitutes a neoplasm derived from B germinal center lymphocytes at different stages of development (1, 2). Its histopathology is characterized by a scarce tumoral cell population (1-10%), termed Hodgkin Reed-Sternberg (HRS) cells, embedded in a rich tumoral microenvironment (TME) composed of B lymphocytes, T lymphocytes, neutrophils, eosinophils, macrophages, plasma cells and histiocytes (3).

The diagnosis of cHL relies on the visual examination of well-obtained, fixed and stained lymph node samples according to the World Health Organization (WHO) criteria for the diagnosis of lymphoid neoplasms (4). Recently, an overview of the upcoming 5^th^ WHO Classification of Haematolymphoid Tumours focusing on lymphoid neoplasms has been published (5), and the essential criteria for the routine diagnosis of cHL seems to remain unchanged. However, a special focus will be on the potential of TME constituents in modulating the biological and clinical behavior of the disease.

Previous studies have reported the prognostic significance of the different cells surrounding HRS cells within the TME, such as regulatory lymphocytes and cytotoxic T lymphocytes (6), programmed death ligand 1 (PD-L1)^+^ T cells (7), Natural Killer (NK) cells (8), B cells (9, 10), monocytes (11) and macrophages (12, 13), with contradictory results (14), among other cell populations, recently reviewed in Menéndez et al. (15).

Thus, the prominence of the TME in cHL and its crucial role on prognosis prompts the need for the adoption and implementation of quantitative, reproducible, precise and highly scalable approaches to study the histopathology of cHL. Regarding this, whole-slide imaging (WSI) and digital image analysis tools represent an opportunity to develop a real TME-based risk stratification model for cHL patients. This aspect constitutes an important need in cHL research, as there is still a fraction (10-20%) of patients who are either refractory or relapse (R/R) after achieving a complete metabolic response (CMR) using current multimodal chemotherapy (16).

However, the literature on the applications of WSI to identify prognostic biomarkers in cHL TME is very scarce, but pioneering studies have reported interesting results. Schäfer et al. described the first pipeline to handle and explore image data of stained cHL tissue images (17). The same group also reported the localization of CD30+ cells in terms of cell graphs by bringing together the fields of digital imaging and network analysis (18). Lately, Hannig et al. proved that HRS cells have neighborhood preferences depending on morphological parameters (19) and, in a recent paper, Jachimowicz et al. (20) demonstrated that a low B-cell content quantified by WSI analysis is a predictor of adverse outcome in patients with advanced-stage cHL treated with BEACOPP (Bleomycin, Etoposide, Adriamycin, Cyclophosphamide, Vincristine, Procarbazine and Prednisone).

In this paper, we perform WSI and digital image analysis in diagnostic formalin-fixed paraffin-embedded (FFPE) lymph node samples of cHL. All patients received the same first-line chemotherapy schedule with Adriamycin, Bleomycin, Vinblastine, Dacarbazine (ABVD) as it constitutes the current standard first-line chemotherapy in cHL (16) and to avoid bias due to the type of treatment received. For further assessing the prognostic significance of the quantified B cells (CD20), T cells (CD3) and HRS cells (CD30), survival analysis was performed using the overall survival (OS) and progression-free survival (PFS) as clinical endpoints.

## Material and methods

### Patients and samples

Formalin-fixed paraffin-embedded (FFPE) lymph node specimens from 85 patients diagnosed with cHL at the Pathology Department of the Puerta del Mar University Hospital and the Puerto Real University Hospital between the years 2009 and 2020 were selected for the study. All the cases satisfied the following criteria: primary diagnosis of cHL, patients’ candidates for intensive treatment, first-line chemotherapy with the ABVD regimen with or without the addition of radiotherapy and immunohistochemical evaluation of B cells (CD20), T cells (CD3) and HRS cells (CD30) in the diagnostic sample. The diagnoses were made in accordance with the 2016 WHO criteria for the diagnosis of lymphoid neoplasms (4). The collection of clinical data was performed from the electronic health record of the patients. We evaluated nine clinicopathological variables in each case: sex, age, histological subtype, Ann Arbor stage with Cotswold´s modifications, presence of B symptoms at diagnosis (fever, drenching night sweats or loss of more than 10% of body weight over 6 months prior to diagnosis), bulky disease (mass in the chest that is one-third the width of the chest, or any lymph node mass greater than 10 cm) and percentage of B cells, T cells and HRS cells in diagnostic samples. Two prognostic variables were recorded: the International Prognostic Score (IPS), categorized as low risk (0-2) and high risk (3-7) (21), and the German Hodgkin Study Group (GHSG) score, categorized as limited, intermediate and advanced stages (22). One variable of response to therapy, measured as the percentage of patients with R/R disease, was also collected. All the samples and data were obtained following the technical and ethical procedures of the local institutions and in accordance with the Helsinki Declaration. The study was approved by the local Ethics Committee (protocol code 1167-N-21).

### Whole-slide image analysis

Tissue specimens for each patient were cut and stained using anti-CD3 (clone SP7, Ventana Medical Systems, Roche), anti-CD20 (clone L26, Ventana Medical Systems, Roche) and anti-CD30 (clone Ber-H2,Ventana Medical Systems, Roche) in the Pathology departments of the participating hospitals. Subsequently, scanning of the slides was centralized at the Puerta del Mar University Hospital (Cadiz, Spain). The PANNORAMIC ^®^ 250 Flash III DX scanner (3D Histech Ltd., Budapest, Hungary) was used to perform the scanning of tissue slides with a resolution of 0.25 microns per pixel, obtaining digital slides in MRXS file format, according to the manufacturer’s recommendations. SlideViewer 2.5 software (3D Histech Ltd., Budapest, Hungary) was used to review the digital slides.

The 3DHistech QuantCenter application (version 2.3) was used for the quantification of CD3, CD20 and CD30 positive cells. Cell quantification was performed in the whole lymph-node specimen, excluding unstained areas and artifacts. Consequently, cell quantification was not restricted to areas of interest within the specimen, and data obtained reflect the whole composition of the diagnostic samples. See the **Supplementary Material** for a detailed description of the WSI and digital image analysis procedures (sections *Digital Image Analysis* and *Reproducibility of image analysis*).

### Statistics

Descriptive statistics was performed for quantitative and qualitative variables. Data are shown as mean (standard deviation, SD) and 95% confidence interval (CI). Normality was assessed by using the Kolmogorov-Smirnov test. For the comparison between qualitative variables, the chi-squared (χ^2^) test was used. Correlation analysis between B cells, T cells and HRS cells was performed by means of the Spearman’s correlation coefficient. Receiver Operating Characteristics (ROC) curves were employed to identify the cut-off value for comparing survival distributions. Survival analysis was performed by using the Kaplan-Meier method with the log-rank test. The OS and the PFS were used as clinical endpoints. All *P*-values were two-sided, and a level of probability below 0.05 was considered significant. The IBM SPSS software (version 15.0) (SPSS Inc., Chicago, IL, USA) and R package (version 4.1.3) were used to perform the statistical analysis. A detailed description of the statistics, clinical endpoints and criteria employed for the evaluation of the response to the therapy (23) are indicated in the **Supplementary Material** (section *Statistics*).

## Results

### Assessment of the cellular composition in classic Hodgkin lymphoma through whole-slide image analysis

**Table 1** shows the clinical, histopathological and prognostic characteristics of the patients included in the study. 58.82% of the patients were male and the mean age of the study cohort was 41.02 years old (SD: 16,36). The most frequent histological subtype was nodular sclerosis (NS) cHL (64.70%) and most of the patients included in the study presented with Ann Arbor stages II (41.17%) and IV (40.00%). Regarding the risk stratification, 51.76% of the cases had a high-risk IPS and 70.58% presented in advanced stages of the GHSG score. As it is common with standard risk-adjusted chemotherapy, most of the patients achieved a CMR with the first-line regimen (77.64%).

**Table 1:** Characteristics of the patients included in the study.

Characteristics (n=85) (%)					
**Sex**	Male Female	***n* =** 50 (58.82%) ***n* =** 35 (41.17%)	**Bulky disease**	Present Absent	***n* =** 11 (12.94%) ***n =*** 74 (87.05%)
**Age**	Mean < 45 ≥ 45	41.02 years old ***n* =** 52 (61.17%) ***n* =** 33 (38.82%)	**EBV-LMP1**	Positive Negative Not performed	***n* =** 24 (28.23%) ***n* =** 27 (31.76%) ***n* =** 34 (40.00%)
**Histological subtype**	NS MC LR NOS	***n* =** 55 (64.70%) ***n* =** 15 (17.64%) ***n* =** 11 (12.94%) ***n* =** 4 (4.70%)	**IPS**	Low risk (0-2) High risk (3-7)	***n* =** 41 (48.23%) ***n* =** 44 (51.76%)
**Ann Arbor stage**	I II III IV	***n* =** 6 (7.05%) ***n* =** 35 (41.17%) ***n* =** 10 (11.76%) ***n* =** 34 (40.00%)	**GHSG**	Limited stages Intermediate stages Advanced stages	***n* =** 5 (5.88%) ***n* =** 20 (23.52%) ***n* =** 60 (70.58%)
**B symptoms**	Present Absent	***n* =** 50 (58.82%) ***n* =** 35 (41.17%)	**Response to first-line therapy**	Complete remission Refractory/relapsed	***n* =** 66 (77.64%) ***n* =** 19 (22.35%)

NS, Nodular Sclerosis; MC, Mixed Cellularity; LR, Lymphocyte-rich; NOS; Not otherwise specified; EBV-LMP1, Epstein-Barr Virus Latent Membrane Protein 1; IPS, International Prognostic Score; GHSG, German Hodgkin Study Group.

**Figure 1:**
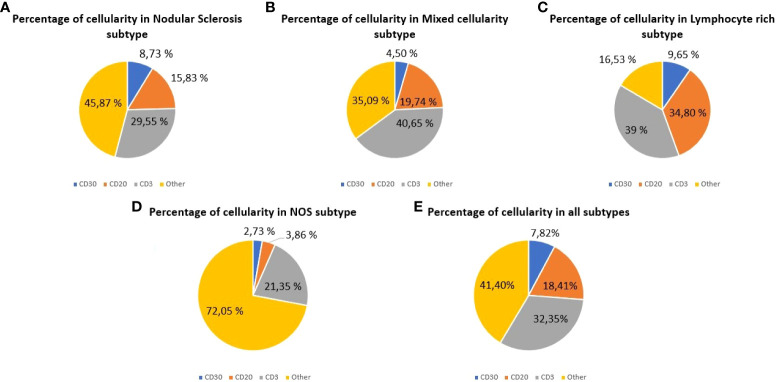
Cellular composition of classic Hodgkin lymphoma (cHL) assessed by whole-slide imaging. Cell percentages for HRS cells (CD30), B cells (CD20) and T cells (CD3). **(A)** Cases of nodular sclerosis cHL. **(B)** Cases of mixed cellularity cHL. **(C)** Cases of lymphocyte-rich cHL. **(D)** Cases of cHL Not Otherwise Specified. **(E)** All the cases analyzed.

Because of one CD3, CD20 and CD30 slides were quantified for each patient, a total of 255 tissue slides were obtained for digitization and analysis. The mean quantified area was 307.92 mm^2^ (SD: 4.94), and the mean total cell count was 1,332,701.30 (SD: 544,407.35) for all the specimens analyzed. The mean percentage of quantified T cells, B cells and HRS cells were 32.35% (95% CI: 28.89-35.82), 18.41% (95% CI: 14.9-21.92) and 7.82% (95% CI: 5.25-10.40), respectively. **Figure 1** shows the relative proportion of B cells, T cells and HRS cells according to the histological subtype.

Data obtained from WSI analysis reveal the cellular heterogeneity for each histological subtype. The sum of B cells and T cells in mixed cellularity (MC) subtype (60.39%) was higher than in NS (45.38%), while HRS cells were more abundant in NS (8.73%) than in MC (4.50%). In the lymphocyte-rich (LR) cHL, the total content of B cells and T cells (73.80%) was higher than in the other subtypes analyzed.

In the bivariate analysis, the histological subtype and the B-cell content were statistically significantly associated (*P*=0.005). Most of the patients with NS subtype (70.90%) presented with a B-cell content below the mean (mean B-cell content: 18.41%; SD: 16.26), while the 72.72% and 60.00% of the cases with LR and MC subtypes, respectively, showed a B-cell content above the mean value. Additionally, the anatomic extension of the disease (Ann Arbor staging system) and the T cell content were also statistically significantly associated (*P*=0.031) and subgroup analysis revealed that the proportion of T cells in the diagnostic samples decreased as the extension of the disease increased. For initial stages (I and II), the 83.33% and the 57.14% of the patients presented with a T cell count above the mean (mean T-cell content: 2.35%; SD: 16.05). Analogously, in advanced stages (III and IV) the 50.00% and the 29.41% of the patients, respectively, presented with a T cell content under the mean value.

**Table 2** shows the results of the correlation analysis. A statistically significant correlation was observed for the comparison between the CD3 and CD20 cell contents (*P*=0.000; ρ=0.423). No statistically significant results were obtained neither for the association between the CD30 and CD3 cell contents (*P*=0.525; ρ=0.070) nor for the association between CD30 and CD20 cells (*P*=0.712; ρ=0.041).

**Table 2:** Results of the correlation analysis between CD30,CD20 and CD3 cell percentages quantified by whole-slide imaging in classic Hodgkin lymphoma.

	CD30 positive cell percentage (Correlation coefficient/*P*)	CD20 positive cell percentage (Correlation coefficient/*P*)	CD3 positive cell percentage (Correlation coefficient/*P*)
**CD30 positive cell percentage**	1	0.041 (*P* = 0.712)	0.070 (*P* = 0.525)
**CD20 positive cell percentage**	0.041 (*P* = 0.712)	1	**0.423** (*P = 0.000)*
**CD3 positive cell percentage**	0.070 (*P* = 0.525)	**0.423** (*P = 0.000)*	1

Boldface indicates statistical significance (P <0.05) by means of the Spearman´s correlation test.

### The percentage of Hodgkin Reed-Sternberg cells impacts survival in patients with classic Hodgkin lymphoma treated with ABVD

After cell quantification, we assessed the prognostic significance of B cells, T cells and HRS cells. When comparing patients with cHL achieving CMR and patients with R/R disease, B-cell content was not associated with a different survival distribution, neither for the OS (*P*=0.295) nor for the PFS (*P*=0.220).

**Figure 2:**
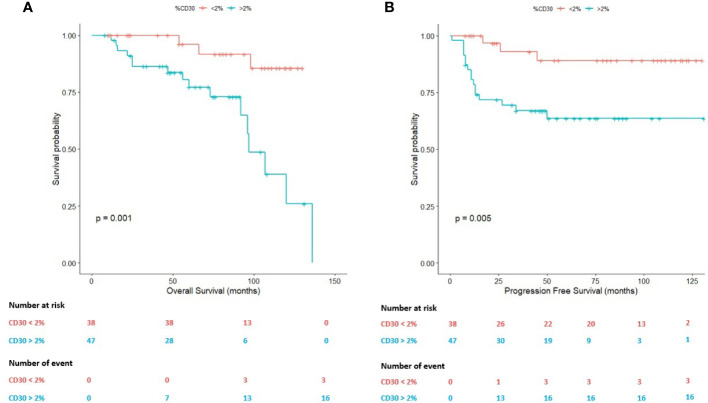
Survival plots (Kaplan-Meier) according to the percentage (2.00%) of Hodgkin Reed-Sternberg (HRS) cells assessed by whole-slide imaging. **(A)** Survival plot for the overall survival (OS). **(B)** Survival plot for the progression-free survival (PFS).

ROC analysis allowed us to identify a 2.00% of HRS cells as an adequate predictor of survival (Area Under the Curve, AUC=0.624). Using this value as cut-off, both OS (*P*=0.001) and PFS (*P*=0.005) statistically significantly differed between R/R patients and patients achieving CMR (**Figure 2**).

On the one hand, the mean OS in cases with a percentage of HRS cells above 2.00% was 95.06 months (95% CI: 80.43-109.70 months) in comparison with patients below this threshold (mean OS: 122.21 months; 95% CI: 113.83-130.59 months). On the other hand, the mean PFS in cases with a percentage of HRS cells above 2.00% was 89.15 months (95% CI: 72.58-105.72 months) in comparison with patients below this cell content (mean PFS:119.07 months; 95% CI: 107.35-130.79 months).

### A low T-cell content quantified through whole-slide imaging confers an adverse prognosis in classic Hodgkin lymphoma

**Figure 3:**
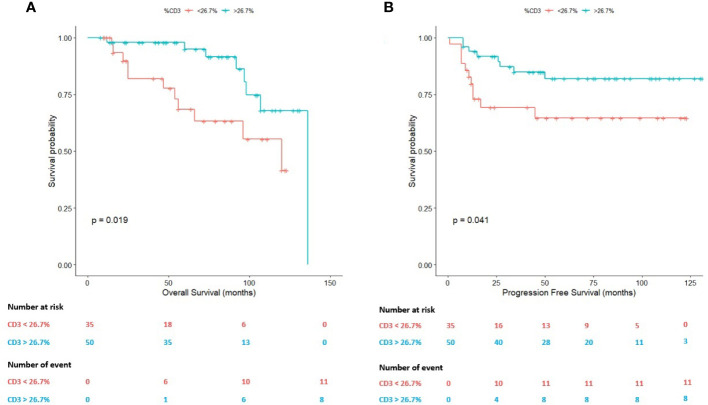
Survival plots (Kaplan-Meier) according to the percentage (26.70%) of T cells assessed by whole-slide imaging. **(A)** Survival plot for the overall survival (OS). **(B)** Survival plot for the progression-free survival (PFS).

Within the cHL TME, T cells seem to modulate the response to therapy (6). A 26.70% of CD3-positive T lymphocytes was identified as an adequate predictor of survival in these patients (AUC=0.531). Using this value as cut-off, both the OS (*P*=0.019) and the PFS (*P*=0.041) statistically significantly differed between R/R patients and patients achieving CMR (**Figure 3**).

On the one hand, the mean OS in cases with a percentage of T cells above 26.70% was 120.26 months (95% CI: 109.42-131.04 months) in comparison with patients below this value (mean OS: 89.52 months; 95% CI: 73.28-105.75 months).

On the other hand, in terms of the PFS, the mean PFS of the patients with a T-cell content above 26.70% was 111.64 months (95% CI: 99.42-123.87 months) in comparison with patients below this value in the diagnostic sample (mean PFS: 84.62 months; 95% CI: 66.01-103.23 months).

### Combining CD30 and CD3 cell contents identify a high-risk subgroup of patients with classic Hodgkin lymphoma

Based on the previous results (subsections 3.2 and 3.3), we hypothesized that the combined impact of HRS cells and T cells could serve to define a high-risk subgroup of patients with cHL. We, thus, assessed the joint impact of CD30-positive HRS cells and CD3-positive T lymphocytes on the survival times.

A priori, the stratification of the study cohort in four different subgroups should reveal differences in long term survival. We classified all the patients (n=85) in four subgroups: 1) Subgroup 1 (S_1_): CD30>2.00% and CD3>26.70% (n=29); 2) Subgroup 2 (S_2_): CD30<2.00% and CD3<26.70% (n=17); 3) Subgroup 3 (S_3_): CD30>2.00% and CD3<26.70% (n=18); 4) Subgroup 4 (S_4_): CD30<2.00% and CD3>26.70% (n=21).

**Figure 4:**
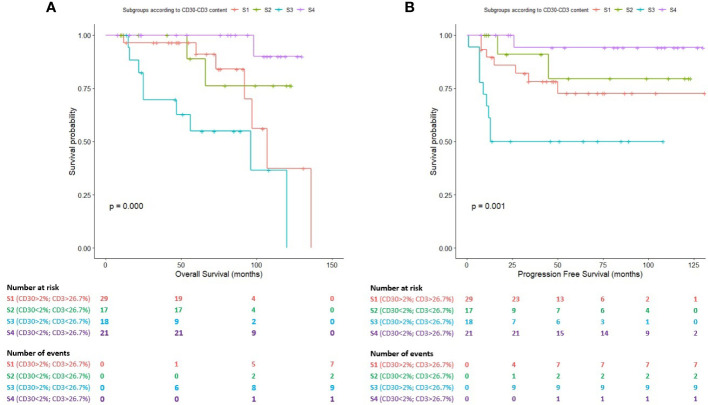
Survival plots (Kaplan-Meier) for subgroups S_1_, S_2_, S_3_ and S_4_ according to the combined impact of HRS cells (2.00%) and T cells (26.70%) assessed by whole-slide imaging. **(A)** Survival plot for the overall survival (OS). **(B)** Survival plot for the progression-free survival (PFS).

**Figure 4** shows the survival distributions for the four subgroups. Patients included in the S_4_ exhibited the longest survival times, whereas patients included in the S3 showed the most unfavorable clinical course. Differences were statistically significant, both in terms of the OS (*P*=0.000; **Figure 4A**) and the PFS (*P*=0.001; **Figure 4B**).

The mean OS for patients included in the S_3_ (high-risk subgroup) was 75.41 months (95% CI: 52.26-98.55 months) in comparison with patients belonging to S_1_ (mean OS: 106.04 months; 95% CI: 87.85-124.22 months), S_2_ (mean OS: 108.09 months; 95% CI: 89.96-126.22 months) and S_4_ (mean OS: 126.80 months; 95% CI:120.85-132.75 months).

The mean PFS for patients included in the S_3_ was 58.44 months (95% CI: 35.52-81.36 months) in comparison with patients belonging to S_1_ (mean OS: 101.70 months; 95% CI: 82.92-120.47 months), S_2_ (mean OS: 104.5 months; 95% CI: 81.44-127.55 months) and S_4_ (mean OS: 123.88 months; 95% CI: 112.25-135.51 months). Cases included in the S_1_ and S_2_ subgroups showed an intermediate clinical course.

## Discussion

Classic Hodgkin lymphoma (cHL) constitutes a model of study to investigate the role of the TME in cancer development and progression. Tumoral HRS cells constitute a minority within the lymph nodes involved by cHL, diluted in a heterogeneous population of B cells, T cells, macrophages, eosinophils, fibroblasts and histiocytes. The different cell types of the TME do not represent an ineffective immunological response, as traditionally considered, but an active partner in cHL pathogenesis, conferring protection to HRS cells within a privileged niche. A topological organization for HRS cells and the cell elements integrating the cHL TME has been previously described (22), supporting the comprehension of cancer as a Darwinian process (24, 25) where the surrounding cells integrating the TME play a fundamental role (11).

In this context, the study of cHL requires a quantitative approach. Features such as intercellular distances and morphological parameters (shape and eccentricity of HRS cells) have been demonstrated to influence its localization within the involved lymph nodes (19). In addition, the principles of network theory have been applied to construct CD30+ cell graphs in cHL tissue (18). However, the transition from these results to the identification of biomarkers with prognostic interest in the management of cHL patients is still lacking. Clinicians (hematologists and oncologists) largely define risk subgroups of patients with cHL based on the GHSG (21) and IPS (26) scores, among others. The parameters considered in these models are mainly clinical, analytical and related to imaging methods, but molecular aspects of the disease are absent. Moreover, the prognostic significance of the components of the TME (15) is not taken into consideration.

In this milieu, where quantification, reproducibility, easy scalability and affordability of the methods are essential for its successful implementation in the departments of Pathology, WSI could significantly contribute to the development of new prognostic models in cHL. The present study aimed to apply WSI and digital image analysis to evaluate a series of cases of cHL and investigate the prognostic significance of the histopathological data obtained.

A previous work conducted by the GHSG had identified a low B cell content by means of WSI as a predictor of adverse outcome in advanced stage-cHL patients treated with BEACOPP (20). We did not identify a prognostic role for B cells surrounding HRS cells neither for the OS nor for PFS. The reasons for this variability could be explained in terms of sample size, but importantly all the patients analyzed in this study received ABVD as standard first-line chemotherapy, which constitutes an important divergence. The literature on this topic is controversial. Some authors have linked B cell abundance with better outcomes (9), but positive cells were counted across 10 to 20 high-powered fields per patient, and not assessing the whole slide. Numerically, we obtained a mean CD20 cell content of 18.41%, for all the specimens, in comparison with a 16.31% for the above-mentioned study. Overall, the mean total cell count quantified by WSI in our study cohort was 1,332,701.30 cells (SD: 544,407.35), in comparison with 1,550,980 (SD: 949,004) for the above-mentioned study. Biologically, there exist arguments to support both a protective and an adverse impact of B cells on the response to therapy in cHL. As stated by Cirillo et al. (27), anti-tumor activity of B-cells in cHL could also reflect remnants of pre-existing lymph nodes indicating just a proxy of a less aggressive disease with a better preservation of the architecture of the lymph node. Future studies would be required to clarify the precise significance of B cells integrating the TME in the natural history of the disease.

The population of T cells constitute the most abundant cell type in the cHL TME (28). We also analyzed its prognostic significance after digitization and quantification of CD3-positive T cells. In a previous study (20), a 53.52% of the total mass of the lymph node has been reported to be constituted by T cells (CD3) (20). We quantified a mean of 32.35% of T cells (SD: 16.05) considering all the specimens analyzed. Interestingly, a 26.70% of T cells accurately discriminates between patients achieving CMR and R/R patients, both for OS and PFS in a statistically significantly manner. This result is consistent with previous studies (6, 29, 30), but contrasts with others (20) that have not reported an association between T cells and outcomes. In a previous study (6), a low infiltration of T-reg cells and a high proportion of cytotoxic T lymphocytes identified through TIA-1 in diagnostic lymph node samples of cHL patients was associated with decreased disease-free survival (DFS) and even-free survival (EFS). Furthermore, the work by Alonso-Alvarez et al. (30) showed that in diagnostic biopsies of cHL patients a high proportion of CD8 T lymphocytes (≥15%) and CD4 T lymphocytes (≥75%) significatively correlated with a decreased 10-year freedom from treatment failure. On the other hand, the work by Jachimowicz et al. (20) did not show an association between the percentage of T cells (CD3) and clinical outcomes of cHL patients, neither for OS nor for PFS. The reasons that could potentially explain the discrepancies observed are multiple, including the techniques used to identify the cell populations (immunohistochemistry, flow cytometry or the combination of immunohistochemistry and digital image analysis), the cell type analyzed (regulatory T cells, cytotoxic T lymphocytes identified by TIA-1, T helper cells identified by CD4, cytotoxic T lymphocytes identified by CD8 or, in general, T population identified by CD3), the lack of homogeneity in relation to the chemotherapy regimens of the patients included in the studies (ABVD, BEACOPP, or others) and, finally, the clinical endpoints selected to perform the survival analysis and the power of the different statistical methods used.

Importantly, the quantification of T cells by means of WSI, using antibodies directed against the CD3 molecule, could be added as a prognostic biomarker in future cHL risk models, as its determination in Pathology departments is not expensive and CD3 constitutes a widely adopted immunohistochemical marker in routine histopathological diagnosis of lymphoid neoplasms. Particularly, in cHL also serves to visualize T cell rosettes which constitute a hallmark of the disease.

However, there exist an enormous heterogeneity in the subsets of T cells existing within the cHL TME: CD4+ T cells (which are the most abundant), CD8+ T cells, central memory T cells, naive T cells, Th1 cells, Th17 cells, among others. Moreover, its impact on the response to treatment seems to be dependent not only on the cell number, but also on their development stages (15). Regarding this, a previous work showed that different subsets of T cells surrounding HRS cells coexist with PD-L1+ tumor associated macrophages (TAMs) within an immunoprivileged niche, promoting HRS cells survival (22). In this work, only two geographically distinct regions were selected for each cHL tumor, and combination with whole slide studies could render important results.

The integration of functional studies with WSI would, thus, give us a more comprehensive view of the clinical significance of T cells in the natural history of cHL. Recently, our group proposed the adoption of a minimum set of genes recurrently mutated in cHL for Next-Generation Sequencing (NGS) studies in cHL tissue (31), and the integration of NGS data with WSI information is the subject of ongoing research in our department.

Finally, we were interested in the assessment of the prognostic significance of the number of HRS cells. Traditionally, the number of HRS cells has not been associated with clinical outcomes (11, 32), and its limited presence within the involved lymph nodes argues for a relative importance on the variability of treatment response. In a previous study (20) the number of HRS cells was not associated with survival times. When quantified, CD30+ HRS cells were counted using the same method based on cell counts than B cells and T cells, which contrast with the method based on area employed in the above-mentioned study. This technical difference could explain the variability to some extent, but chemotherapy regimens, sample size and demographics of the patients also contribute importantly to the heterogeneity in this point.

It is remarkable that previous studies performed on the modern era of the multimodal chemotherapy in cHL have demonstrated that the use of risk-adjusted protocols has abolished the prognostic role of histopathological data (33, 34). This fact contrasts with the crescent evidence supporting that histopathology of the TME results essential to comprehend the natural history and the response to treatment of the disease (6–8, 12, 13, 15). By using WSI and digital image analysis, there is the need for larger studies to compare results and clarify if the histopathological features of the tumor and, particularly, the number of HRS cells in lymph nodes involved by cHL influences the clinical course of the disease.

Despite the results provided in the present paper applying WSI analysis to identify prognostic biomarkers in cHL TME could be of interest, several limitations must be addressed. First, we have used single chromogenic immunomarkers for identifying cell populations. This approach is limiting the assessment of cellular subtypes that can be identified, and it would be of interest to incorporate multiple markers to better typify the T cell population (i.e. T-regulatory cells). The design of this approach requires to properly define the cellular structures which will be stained in order to not invalidate the evaluation of the obtained results, as CD20, CD30 and CD3 are membrane-localized structures that could not be easily combined in a single multiple labeling panel. Second, we only considered cHL patients treated with ABVD. This point could reduce the variability due to the inclusion of cHL patients treated with different chemotherapy schemes (ABVD, BEACOPP, among others), however, it also limits the generalizability of the results to cHL patients that receive treatment other than ABVD.

Tumor microenvironment in cHL accounts for the main volume of the tumor mass. Considering the relevance of clinical translation and specifically looking for the identification of prognostic biomarkers in the TME, we are in accordance with Jachimowicz et al. (20) in that WSI presents the potential, affordability and easy scalability needed for a successful implementation in future cHL clinical trials. Novel technological advantages (35) and the combination of digital image analysis with NGS data could contribute to better comprehend both the molecular pathogenesis of cHL and the correlation of histopathological and genomic features with the response to treatment.

## Data availability statement

The raw data supporting the conclusions of this article will be made available by the authors, without undue reservation.

## Ethics statement

The studies involving human participants were reviewed and approved by the Puerta del Mar University Hospital Ethics Committee (protocol code 1167-N-21). The patients/participants provided their written informed consent to participate in this study.

## Author contributions

AS-E and IB-F conceived of the experiments; AS-E, IB-F, JP-R, LA-C, and NM-G conducted the experiments and AS-E, IB-F and MG-R analyzed the results. All authors contributed to the article and approved the submitted version.

## Funding

This work was supported by a postdoctoral grant (RH-0145-2020) from the Andalusia Health System and with an EU FEDER ITI Grant for Cadiz Province PI-0032-2017.

## Conflict of interest

The authors declare that the research was conducted in the absence of any commercial or financial relationships that could be construed as a potential conflict of interest.

## Publisher’s note

All claims expressed in this article are solely those of the authors and do not necessarily represent those of their affiliated organizations, or those of the publisher, the editors and the reviewers. Any product that may be evaluated in this article, or claim that may be made by its manufacturer, is not guaranteed or endorsed by the publisher.
