# Supplementary material for: Whole-slide image analysis identifies a high content of Hodgkin Reed-Sternberg cells and a low content of T lymphocytes in tumor microenvironment as predictors of adverse outcome in patients with classic Hodgkin lymphoma treated with ABVD
Source: Front Oncol. 2022 Oct 20;12:1000762. doi: 10.3389/fonc.2022.1000762 (PMC9631766; doi:10.3389/fonc.2022.1000762)
Supplement: Supplementary file 1 [file DataSheet_1.pdf]

Supplementary Material

1 Digital image analysis

The scanning of 255 histopathological slides from 85 patients diagnosed of cHL was performed in the Pathology Department of Puerta del Mar University Hospital (Cadiz, Spain). The PANNORAMIC® 250 Flash III DX scanner (3DHistech Ltd., Budapest, Hungary) was used for the digitization process. The images obtained, with a resolution of 0.25 microns per pixel, were stored in .MRXS file format. File size per image varied from 100 MB to 6.30 GB depending on the scanned tumor size. The visualization of digitized preparations was performed using the SlideViewer 2.5 software (3D Histech Ltd., Budapest, Hungary). The 3DHistech QuantCenter application 2.3 was employed for running inmunohistochemical measurements of anti-CD30 (clone Ber-H2,Ventana Medical Systems, Roche), anti-CD20 (clone L26, Ventana Medical Systems, Roche) and anti-CD3 (clone SP7, Ventana Medical Systems, Roche) antibodies on digital slides. Quantification of CD30+ Hodgkin Reed-Sternberg (HRS) cells, CD20+ B lymphocytes and CD3+ T lymphocytes was performed by using the MembraneQuant analyzing algorithm (magnification 0.2x). The discrimination between CD20+ HRS cells and reactive CD20+ B cells and CD3+ T cells was performed through visual inspection by pathologists from the three participating hospitals (JPR, LAC, NMG and MGR) and based on morphological criteria, as HRS are large cells usually binucleated or multinucleated whereas reactive B lymphocytes and CD3+ T cells constitute populations of small and round nucleated cells. The thresholds values for the antibody detection were established manually at a higher magnification (20x) in order to overcome the problem of different immunostaining intensities for each antibody. Particularly, for the distinction between CD30+ immunoblasts and HRS cells, most of the cases analyzed presented with a diffuse infiltration of the lymph node. In cHL cases with some preserved areas of the lymph node architecture, immunoblasts of the paracortical T zone and interfollicular areas were separated from HRS cells by elimination of these areas after evaluation of haematoxylin-eosin stained tissue by hematopathologists.

The parameters and values established to quantify the number of HRS cells, B cells and T cells with stained membranes are indicated in the Supplementary Table 1. In order to comprehensively evaluate the whole tissue, we performed tissue-background separation, and the whole lymph node specimen was analyzed. For each slide, the QuantCenter algorithm generates a document in CSV (Comma-separated values) format with the following information: annotation area quantified (mm^2), total cell count (absolute value), negative cells (absolute and percentage) and positive cells (absolute and percentage). Data were exported from the CSV document for performing statistical analysis. The supplementary figures 1, 2 and 3 show a cHL tissue specimen for the quantification of HRS cells, B lymphocytes and T lymphocytes, respectively.

2 Reproducibility of image analysis

To evaluate the reproducibility of the digital image analysis procedure, a total of 15 cHL patients included in the study were randomly selected. All cell counts were performed for a second time for CD30 (stained for anti-CD30; clone Ber-H2,Ventana Medical Systems, Roche), CD20 (stained for anti-CD20;clone clone L26, Ventana Medical Systems, Roche) and CD3 (stained for anti-CD3; clone SP7, Ventana Medical Systems, Roche) cell detection by a second member of the research group, blinded for the results of the first analysis.

The reproducibility study was performed, thus, in a total of 45 cHL digital slides and correlation parameters were calculated for each cell population (CD30, CD3 and CD20). The Spearman correlation coefficients were 1 (*P*=0.000) for the comparison between the two quantification analysis for HRS cells, B lymphocytes and T lymphocytes (Supplementary Figure 4).

To validate the results obtained in the survival analysis we performed split-sample and cross-validation techniques. We have performed split-sample and cross-validation methods, defining partitions of 33/67 and 25/75 of the cases for conducting the split-sample method and defining a training set and a validation set in each case. We obtained a statistically significant association between the percentage of CD30+ HRS cells and the OS and PFS both in the training set (OS: P=0.014; PFS: P=0.022) and the validation set (OS: P=0.024, PFS: P=0.013). Percentage of CD20 cells was not associated with clinical outcomes, as in the original dataset. Furthermore, the percentage of CD3+ T cells retained statistical significance only in the training set for the OS (P=0.015) but not for PFS (P>0.05); additionally, both for the OS and PFS the joint impact of the expression of a high-content of CD30+HRS cells and low content of CD3+ T lymphocytes also showed statistical significance for the OS intraining set (P=0.005) and validation set (P=0.049). The PFS was also statistically significatively associated with CD3-CD30 cell content (P=0.000). On the other hand, we have performed cross-validation by means of two techniques: (A) fixing the number of events; and (B) fixing the ratio of events. An event in our study was defined as the presence of relapsed or refractory disease (R/R). First, when fixing the number of events in 10 cases (A) we obtained analogous results for CD30 and CD3-CD30. This effect was particularly evident in the case of n=50 cases (10 events and 40 non-events or patients achieving a complete metabolic response), but also when n=30 (10 events and 20 non-events) and n=70 (10 events and 60 non-events). When the ratio of events was fixed in a proportion of 1:3 to ensure a sufficient sample size (5 events and 10 non-events, ratio 1:3) CD30+ HRS cells retained statistical significance both for OS (P=0.003) and PFS (P=0.006), as CD3 (0S: P=0.006; PFS: P=0.002) and the combination of CD3 and CD30 cells percentages in the diagnostic samples (P=0.000 for OS and PFS).

3 Statistics

The overall survival (OS) and the progression-free survival (PFS) were used as clinical endpoints to perform the survival analysis by means of the Kaplan-Meier method with the log rank test. The OS was defined as the time interval between the initial histopathological diagnosis and death, lost to follow-up or end of the study. The PFS was defined as the time interval between the initial histopathological diagnosis and the first progression or relapse after achieving a complete metabolic remission (CMR). The evaluation of the response to the treatment was performed by positron emission tomography/computed tomography (18) (F-FDG PET/CT) following the revised Cheson criteria (35).

Supplementary Table 1. Parameters established in the QuantCenter algorithm for the quantification of CD30, CD20 and CD3 positive cell populations

**CD30**	**Membrane detection algorithm**
**Filter**	Sensitivity (255)
	Intensity (100-255)
	Area (40-300)
**CD20**	**Membrane detection algorithm**
**Filter**	Sensitivity (230)
	Intensity (0-255)
	Area (40-300)
**CD3**	**Membrane detection algorithm**
**Filter**	Sensitivity (230)
	Intensity (0-255)
	Area (40-300)

4 Supplementary Figures legends

Supplementary Figure 1. Assessment of CD30 cellularity in cHL lymph node. (A). Image of the whole lymph node stained for CD30 (0.4X). (B). A magnified area (10X) of the specimen where HRS cells can be distinguished (stained cells). (C). The same region after cell quantification using the MembraneQuant algorithm (3D Histech Ltd., Budapest, Hungary). Blue, yellow, orange and red colors represent negative, weak, medium and strong cellular positivity, respectively.

Supplementary Figure 2. Assessment of CD20 cellularity in cHL lymph node. (A). Image of the whole lymph node stained for CD20 (0.4X). (B). A magnified area (10X) of the specimen where B cells can be distinguished (stained cells). (C). The same region after cell quantification using the MembraneQuant algorithm (3D Histech Ltd., Budapest, Hungary). Blue, yellow, orange and red colors represent negative, weak, medium and strong cellular positivity, respectively.

Supplementary Figure 3. Assessment of CD3 cellularity in cHL lymph node. (A). Image of the whole lymph node stained for CD3 (0.4X). (B). A magnified area (10X) of the specimen where T cells can be distinguished (stained cells). (C). The same region after cell quantification using the MembraneQuant algorithm (3D Histech Ltd., Budapest, Hungary). Blue, yellow, orange and red colors represent negative, weak, medium and strong cellular positivity, respectively.

Supplementary Figure 4. Dispersion diagrams obtained from the reproducibility analysis. (A). Dispersion diagram for CD30 cell population (HRS cells). (B). Dispersion diagram for CD20 cell population (B cells). (C). Dispersion diagram for CD3 cell population (T cells).
