# Supplementary figures and images for: Whole-slide image analysis identifies a high content of Hodgkin Reed-Sternberg cells and a low content of T lymphocytes in tumor microenvironment as predictors of adverse outcome in patients with classic Hodgkin lymphoma treated with ABVD
Source: Front Oncol. 2022 Oct 20;12:1000762. doi: 10.3389/fonc.2022.1000762 (PMC9631766; doi:10.3389/fonc.2022.1000762)

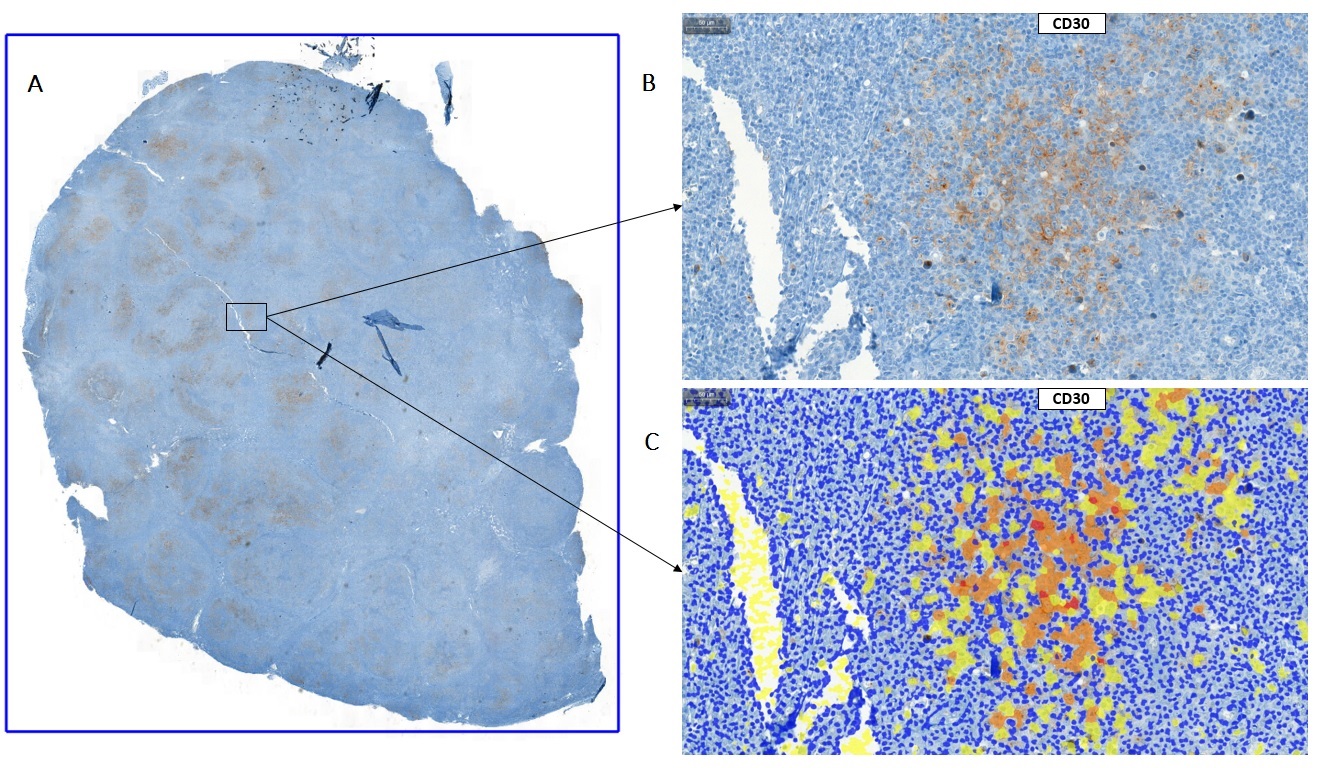

Supplement: Supplementary file 2 [file Image_1.jpeg]

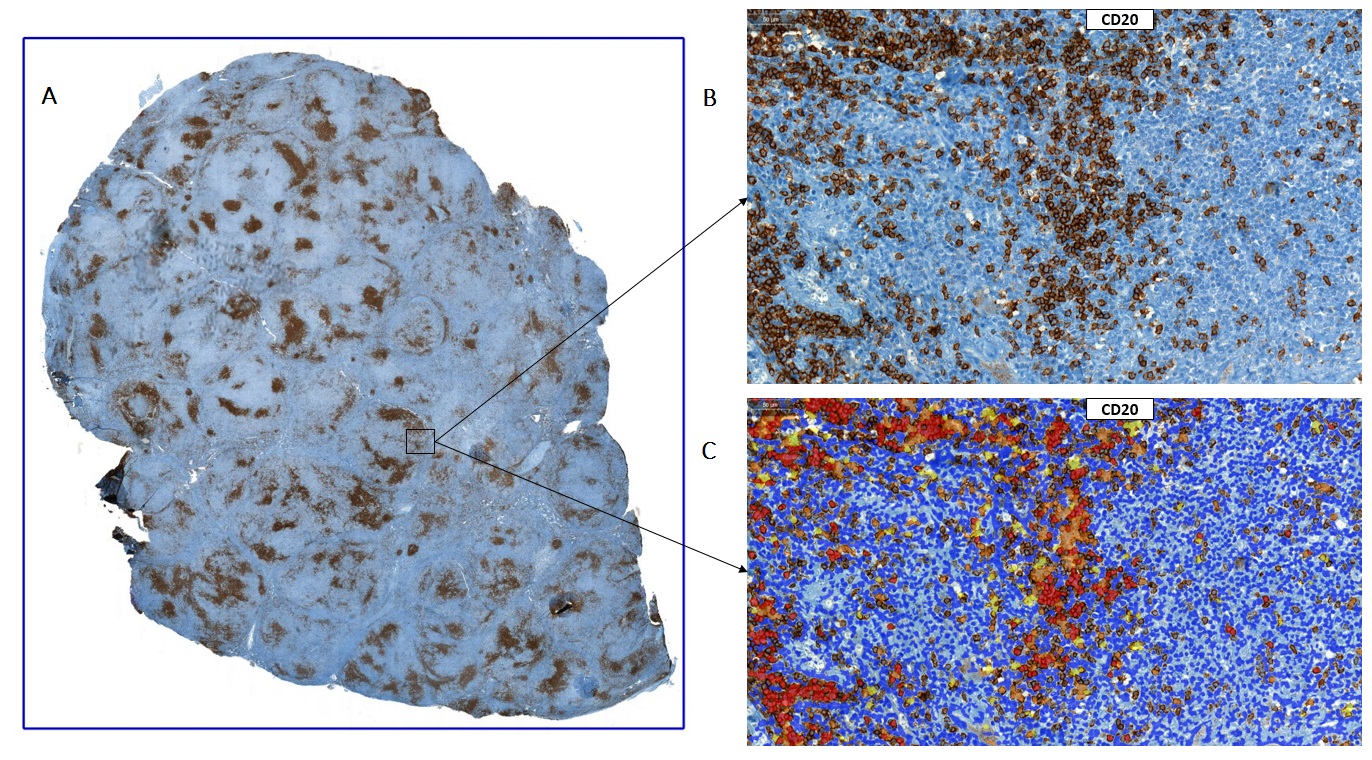

Supplement: Supplementary file 3 [file Image_2.jpeg]

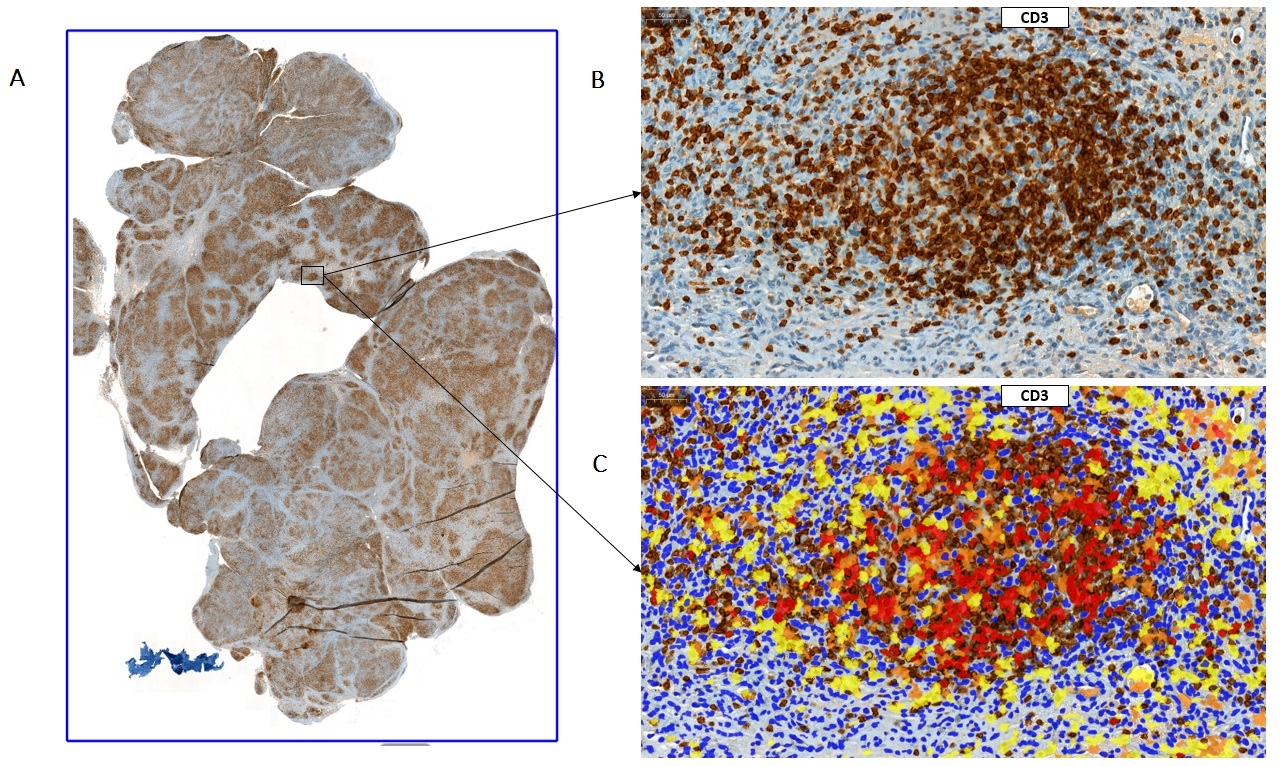

Supplement: Supplementary file 4 [file Image_3.jpeg]

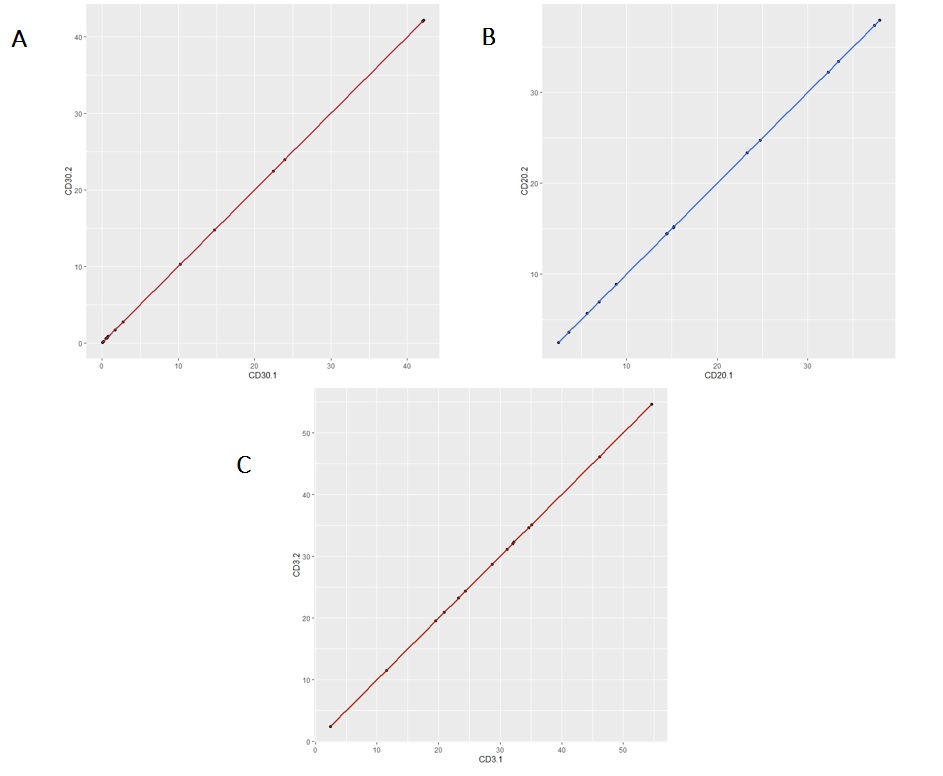

Supplement: Supplementary file 5 [file Image_4.jpeg]
